# My experience with cubital tunnel syndrome

**DOI:** 10.1186/1753-6561-9-S3-A75

**Published:** 2015-05-19

**Authors:** Goo Hyun Baek

**Affiliations:** 1 Department of Orthopaedic Surgery, Seoul National University Hospital, Seoul, 110-744, Korea

## 

Cubital tunnel syndrome can be defined as a compressive neuropathy of the ulnar nerve around the elbow from the arcade of Struthers to the flexor-pronator aponeurosis. However, this complex neuropathy is related not only with *compressive force* but also with *tensile force* caused by elongation of the ulnar nerve while flexing the elbow joint and with *friction force* caused by excursion of the nerve during elbow motion.

## Anatomic considerations

There are five potential sites for the ulnar nerve compression around the elbow - arcade of Struthers, medial intermuscular septum, cubital tunnel retinaculum (Osborne’s ligament), Osborne’s fascia covering two heads of flexor carpi ulnaris, and deep flexor-pronator aponueurosis. Sometimes, the ulnar nerve can be compressed by synovial hypertrophy of the elbow in rheumatoid disease, the presence of the tumors such as ganglion or lipoma, or aberrant muscles such as anconeus epitrochlearis.

**Figure 1:**
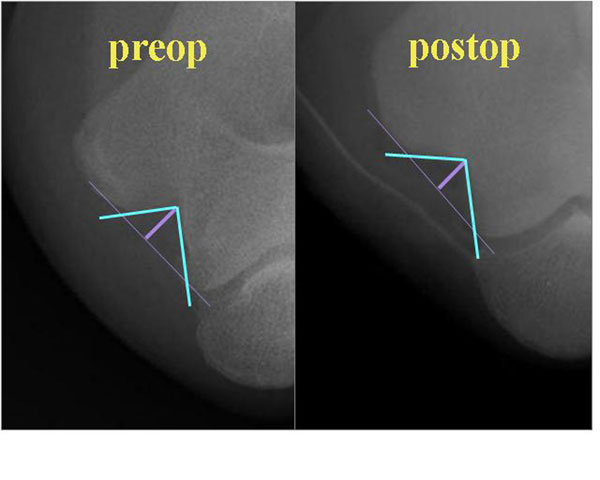
Both cubital tunnel angle (CTA) and cubital tunnel depth (CTD) became wider and shallower after minimal medial epicondylectomy and in situ decompression

Bony structure of the cubital tunnel can be easily seen by a simple X-ray, the cubital tunnel view, which is an AP view with full flexion and 20 degrees of external rotation of the elbow. I proposed two parameters of the cubital tunnel view, cubital tunnel angle (CTA) and cubital tunnel depth (CTD) (Figure 1). When the CTA is smaller, the angle is narrower; or when the CTD is large, the depth is deeper than normal, thus the chance of ulnar nerve irritation at the cubital tunnel will be increased.

The bony anatomy of the cubital tunnel is one of the important etiologic factors in cubital tunnel syndrome, as well as abnormalities of soft tissue anatomy.

## Operative procedures

Initial conservative therapy is widely recommended for mild cases of cubital tunnel syndrome, however, there have been no general agreement on the indications and specific methods of surgery for the more severe cases.

Although there are many operative procedures described for the treatment of cubital tunnel syndrome, these can be categorized into three groups – in situ decompression (open or endoscopic), decompression and anterior transposition (subcutaneous or submuscular), and in situ decompression and medial epicondylectomy.

I prefer in situ decompression when cubital tunnel angle (CTA) and cubital tunnel depth (CTD) are within normal limits. However, when the CTA is narrower and/or CTD is deeper than normal I do minimal medial epicondylectomy as well as in situ decompression.

The main advantage of this technique (in situ decompression + minimal medial epicondylectomy) is to broaden CTA and shorten CTD (Figure). Other advantages of this procedure include preservation of the ulnar nerve blood supply, minimizing trauma to the nerve, smaller incision than anterior transposition technique, and nerve decompression posterior to the medial epicondyle. Although some disadvantages such as loss of the protective prominence of the medial epicondyle, postoperative tenderness at the osteotomy site, and weakness related to detachment of the flexor pronator origin, these complications are very rare in my hand.

## Summary

A good deal of controversy surrounds the natural history, pathogenesis, surgical indications, and methods of surgery for cubital tunnel syndrome. The surgical treatment options for the ulnar nerve entrapment at the elbow include in situ decompression (open or endoscopic), decompression and anterior transposition (subcutaneous or submuscular), and in situ decompression and medial epicondylectomy, all of which are used with relative frequency based on surgeon preference. Considering the importance of bony anatomy of the cubital tunnel such as CTA and CTD, minimal medial epicondylectomy is necessary to be combined with in situ decompression especially when CTA is narrow and/or CTD is deep.
